# cRegulome: an R package for accessing microRNA and transcription factor-gene expression correlations in cancer

**DOI:** 10.7717/peerj.6509

**Published:** 2019-03-08

**Authors:** Mahmoud Ahmed, Deok Ryong Kim

**Affiliations:** Department of Biochemistry and Convergence Medical Sciences, Institute of Health Sciences, Gyeongsang National University School of Medicine, JinJu, GyeongNam, Republic of Korea

**Keywords:** R, R microRNA, Transcription factor, Expression correlation, Cancer, Cistrome

## Abstract

**Background:**

Transcription factors and microRNAs play a critical role in regulating the gene expression in normal physiology and pathological conditions. Many bioinformatics tools were built to predict and identify transcription factor and microRNA targets and their role in the development of diseases including cancers. The availability of public access high-throughput data allows researchers to make data-driven predictions.

**Implementation:**

Here, we developed an R package called cRegulome to access, manage and visualize data from open source databases. The package provides a programmatic access to the regulome (transcription factor and microRNA) expression correlations with target genes of different cancer types. It obtains a local instance of Cistrome Cancer and miRCancerdb databases and provides classes and methods to query, interact with and visualize the correlation data.

**Availability:**

cRegulome is available on the comprehensive R archive network (CRAN) and the source code is hosted on GitHub as part of the ROpenSci on-boarding collection, https://github.com/ropensci/cRegulome.

## Introduction

Transcription factors and microRNAs are important regulators of the gene expression in normal physiology and pathological conditions such as cancer (Bhagwat & Vakoc, 2015; Hayes, Peruzzi & Lawler, 2014). Efforts have been made to build a complete portfolio of transcription factors and microRNAs in different diseases. Cistrome Cancer is a web tool based on an integrative analysis of The Cancer Genome Atlas (TCGA) and ChIP-Seq public data. It describes the expression correlations between hundreds of transcription factors and their target genes in many types of cancer (Mei et al., 2017; Hudson et al., 2010). miRCancerdb is another web tool based on a similar integrative analysis of TCGA and the TargetScan annotations to find the target genes of microRNAs. It’s used to calculate the expression correlations between microRNAs and target genes in various types of cancers (Ahmed et al., 2018b; Hudson et al., 2010; Agarwal et al., 2015). Combination of these two tools offer a systematic way to investigate the role of the transcription factors and microRNAs in the development of cancer.

Multiple resources are available to investigate the regulation of gene expression by means of cis- and trans-elements of the regulome. These particularly interest researchers who wish to conduct genome wide investigation or decipher the role of certain elements alike. Integrating more than one kind of data can be informative (Hamid et al., 2009). In practice, this research can be challenging for several reasons (Kesh & Raghupathi, 2004), because of the different user APIs, user interfaces and naming conventions. Third-party tools can provide access to several databases of a similar nature in unified interfaces. This can facilitate certain types of research, allow for combining data sources and boost the reproducibility of investigations.

Here, we developed a uniform programmatic interface to get and analyze the data from two databases using the popular R language (R Core Team, 2017). cRegulome is an R package that can obtain, manage and visualize regulome expression correlations in TCGA cancer studies. In this article, we introduce the package with examples and a case study from the published literature.

## Materials & Methods

**Figure 1:**
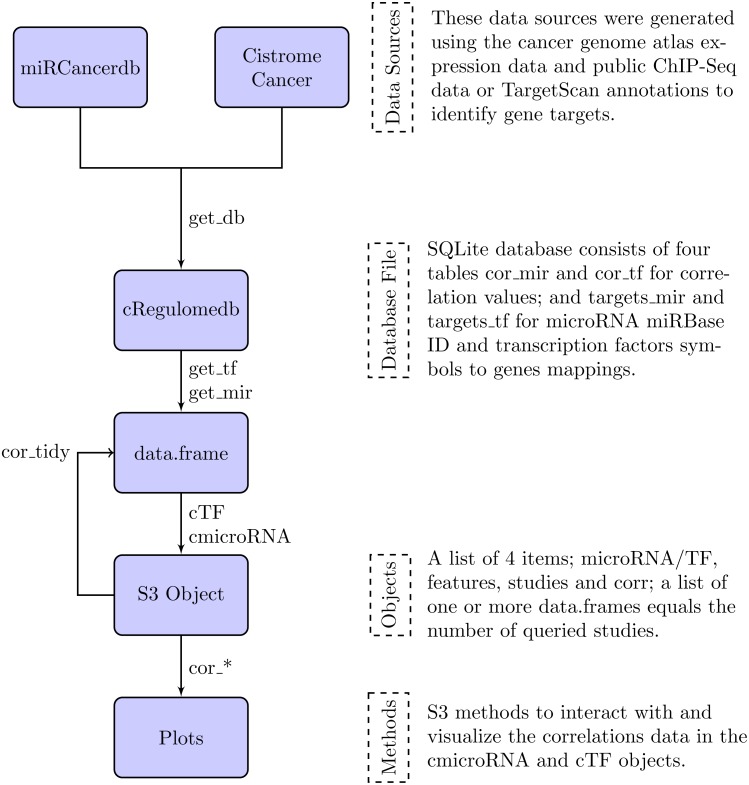
Flowchart describing the implementation of the cRegulome package. The four main components of the package are the data sources, the database file, the objects and the methods. Each is described in text and the specific implementation in the package is illustrated in the flowchart. The links are labeled by the specific function from the package.

In the following section, we describe the data sources that were used to build the database file, and the methods to interact with the database and their outputs. Figure 1 is a summary diagram of the package implementation.

### Data sources

The database file was built based on two data sources: Cistrome Cancer and miRCancerdb databases (Mei et al., 2017; Ahmed et al., 2018b). Based on an integrative analysis of TCGA and public ChIP-seq data, Cistrome Cancer provided the expression correlations of transcription factors (*n* = 305) and their target genes in different cancer types (*n* = 29). In addition, Cistrome Cancer calculated the regulatory potential of transcription factors with target or non-target genes. Similarly, miRCancerdb used TCGA data and TargetScan annotations to determine the expression correlation between microRNAs (*n* = 684) and target or non-target genes in cancer types (*n* = 34).

### Database file

The cRegulome can obtain and query a pre-built SQLite database file of the Cistrome Cancer and miRCancerdb databases. The details of this build were described in cRegulomedb (https://github.com/MahShaaban/cRegulomedb). In addition, this repository contains the scripts and documentation to pull, format and deposit the data at an on-line repository. Briefly, the SQLite database consists of four tables cor_mir and cor_tf for correlation values; and targets_mir and targets_tf for microRNA miRBase ID and transcription factor symbols to gene mapping. Two indices were created to facilitate the database search using the miRBase IDs and transcription factor symbols. The database file can be downloaded using the function get_db.

### Database query

To query the database using cRegulome, we provided two main functions with get_mir and get_tf for microRNA and transcription factor correlations, respectively. Users need to provide the proper IDs for microRNA, transcription factor symbols and/or TCGA study identifiers. microRNAs are referred to by the official miRBase IDs, transcription factors by their corresponding official gene symbols and TCGA studies with their common identifiers. In either cases, the output of calling these functions is a tidy data.frame of four columns; mirna_base or tf, feature, cor and study. They correspond to each of the miRBase IDs or transcription factor coding gene symbols, target gene symbols, correlation values and the TCGA study identifiers.

### Objects

Two S3 (simple storage service) objects are defined in cRegulome to store and dispatch methods on the correlation data: cmicroRNA and cTF for microRNA and transcription factors, respectively. The structure of these objects is very similar. Basically, as all S3 objects, it’s a list of four items; microRNA or TF for the regulome element, features for the gene hits, studies for the TCGA studies and finally corr is a named list of data.frames-one for each queried study. Each of these data.frames has the regulome element (microRNAs or transcription factors) in columns and features/genes in rows. To construct these objects, users need to call a construct function with the corresponding names on the data.frame output form get_*. The reverse is possible by calling the function cor_tidy on the object to get back the tidy data.frame.

### Methods

cRegulome provides S3 methods to interact with and visualize the correlations data in the cmicroRNA and cTF objects. Table 1 provides an overview of these functions. These methods dispatch directly on the objects and could be customized and manipulated in the same way as their generics. Generally, the functions fall in one of two categories. One is for obtaining and querying the database file and another for managing and visualizing the output data.

**Table 1:** Description of the package functions and methods.

Method	Description
get_db	Download the database file.
get_mir	Get correlations of the input microRNA with all genes or targets only.
get_tf	Get correlations of the input TF with all genes or targets only.
cor_tidy	Transform the objects cTF and cmicroRna to a tidy data.frame.
cor_hist	Makes a histogram of each of the regulome elements correlation values.
cor_joy	Makes a joy plot for each of the regulome elements correlation values.
cor_plot	Makes a dot plot of element correlations with colors corresponding to direction and size to the value of the correlation.
cor_upset	Makes an upset plot of the intersections of sets of regulome elements’ feature/gene hits.
cor_venn_diagram	Makes a Venn diagram of the numbers and the intersection of the regulome elements’ feature/gene hits.
cor_igraph	Make a directed graph of the regulators TF/microRNA and their targets.

## Results

### Identifying common regulators of PEBP1 and autophagy/EMT genes

To illustrate the use of the cRegulome package in answering relevant biological questions, we studied candidate transcriptional regulators of RKIP, an anti-cancer gene product known also as phosphatidylethanolamine binding protein (PEBP1) (Ahmed et al., 2018a). In this study, we used the weighted-gene co-expression network analysis (WGCNA) to find the epithelial to mesenchymal transition (EMT) and autophagy gene products that potentially interact with PEBP1 during the development of prostate cancer. These interactions could be either a direct physical binding or an indirect relation. Gene products that share common upstream regulators are expected to show high co-expression at mRNA level. Using the cRegulome package, we identified two transcription factors and three microRNAs that commonly regulate PEBP1 and one or more of the eight gene products of interest.

### Database query and target filtering

We first used the cRegulome to identify transcription factors and microRNAs that target PEBP1 in prostate cancer study (PRAD). Two transcription factors were also found to target one or more of the eight genes of interest and three microRNAs were found to target PEBP1 only (Table 2). The transcription factors were RCC Excision Repair 6, Chromatin Remodeling Factor (ERCC6) and Vascular Endothelial Zinc Finger 1 (VEZF1). The three microRNAs were hsa-mir-23c, hsa-mir-378c and hsa-mir-761. The full list of targets and their co-expression correlations were retained to investigate their global regulatory roles.

**Table 2:** Output of transcription regulators from cRegulome.

Type	Transcriptional regulator	Target	Correlation
miRNA	hsa-mir-23c	PEBP1	−0.16
		PIK3C3	0.23
		TBC1D5	0.34
		TGFBR1	0.23
		TOLLIP	−0.12
		WDR45	−0.22
	hsa-mir-378c	PEBP1	−0.21
		PIK3C3	0.12
		PIK3CB	−0.13
		TBC1D5	0.19
		TGFBR1	0.10
		TOLLIP	−0.22
		WDR45	−0.11
	hsa-mir-761	PEBP1	0.17
		PIK3C3	−0.10
		TOLLIP	0.11
TF	ERCC6	PEBP1	−0.47
		TBC1D5	0.66
	VEZF1	PEBP1	−0.56
		PIK3C3	0.57
		WDR45	−0.51

### Visualization of the query output

**Figure 2:**
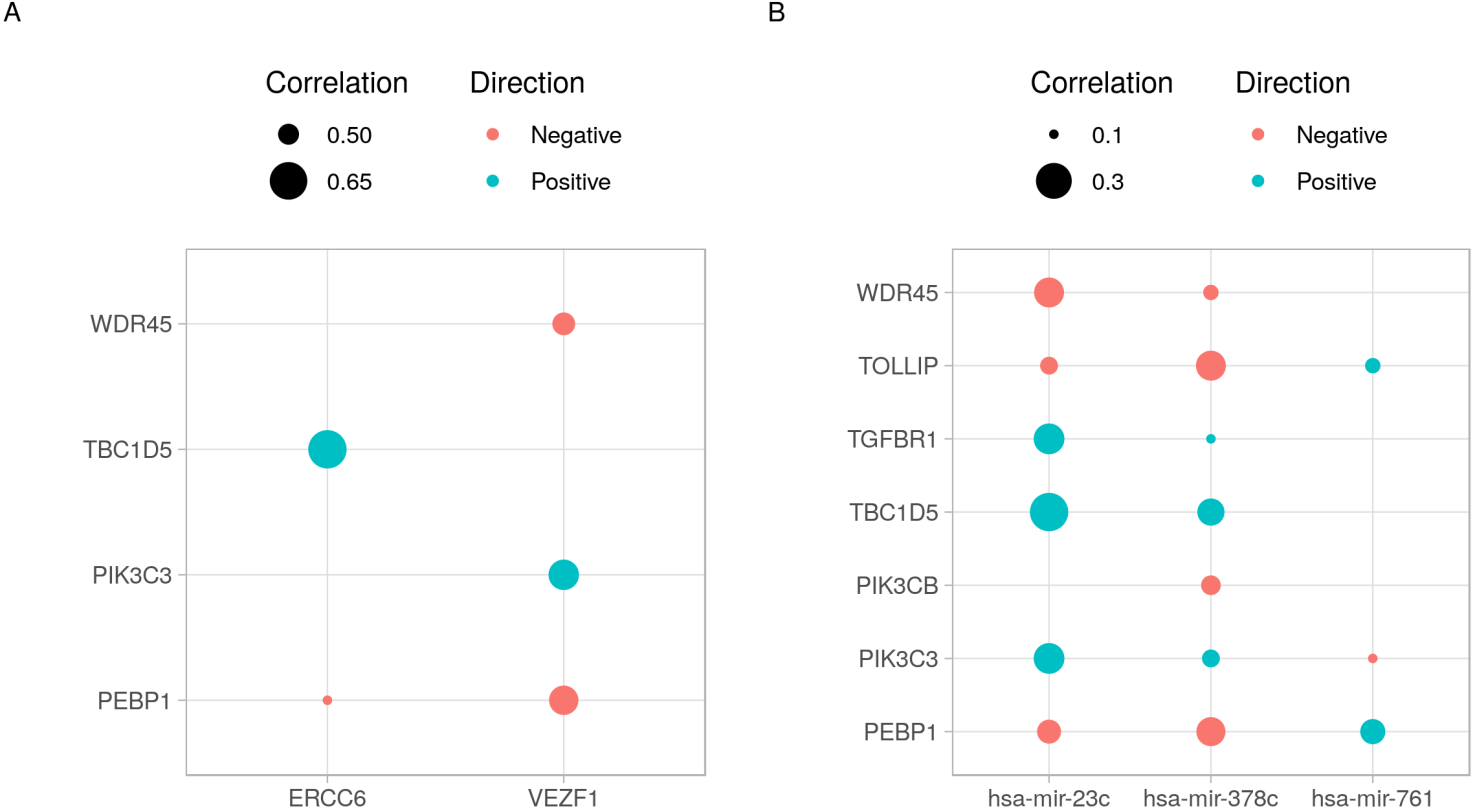
Dot plot of transcription factor/microRNA-gene expression correlations. The expression correlations between target genes and (A) transcription factors or (B) microRNAs are shown as dots. The magnitude of the correlation, when present, is indicated by the size of the dot. The direction of the correlation is indicated by colors; positive (> 0) as blue and negative (< 0) as red.

cRegulome provide a set of functions to visualize the query outputs. Figure 2 is a dot plot of the filtered query. The expression correlations of each regulator are shown as a dot. The size of the dot is the magnitude of the correlation. Two points are immediately obvious in this figure. First, both negative and positive regulations are present for this set of target. Second, the target TBC1D5 has the strongest regulation with most regulators. The same regulatory connection between ERCC6 and TBC1D5 appear in multiple other cancer types (Table S1).

**Figure 3:**
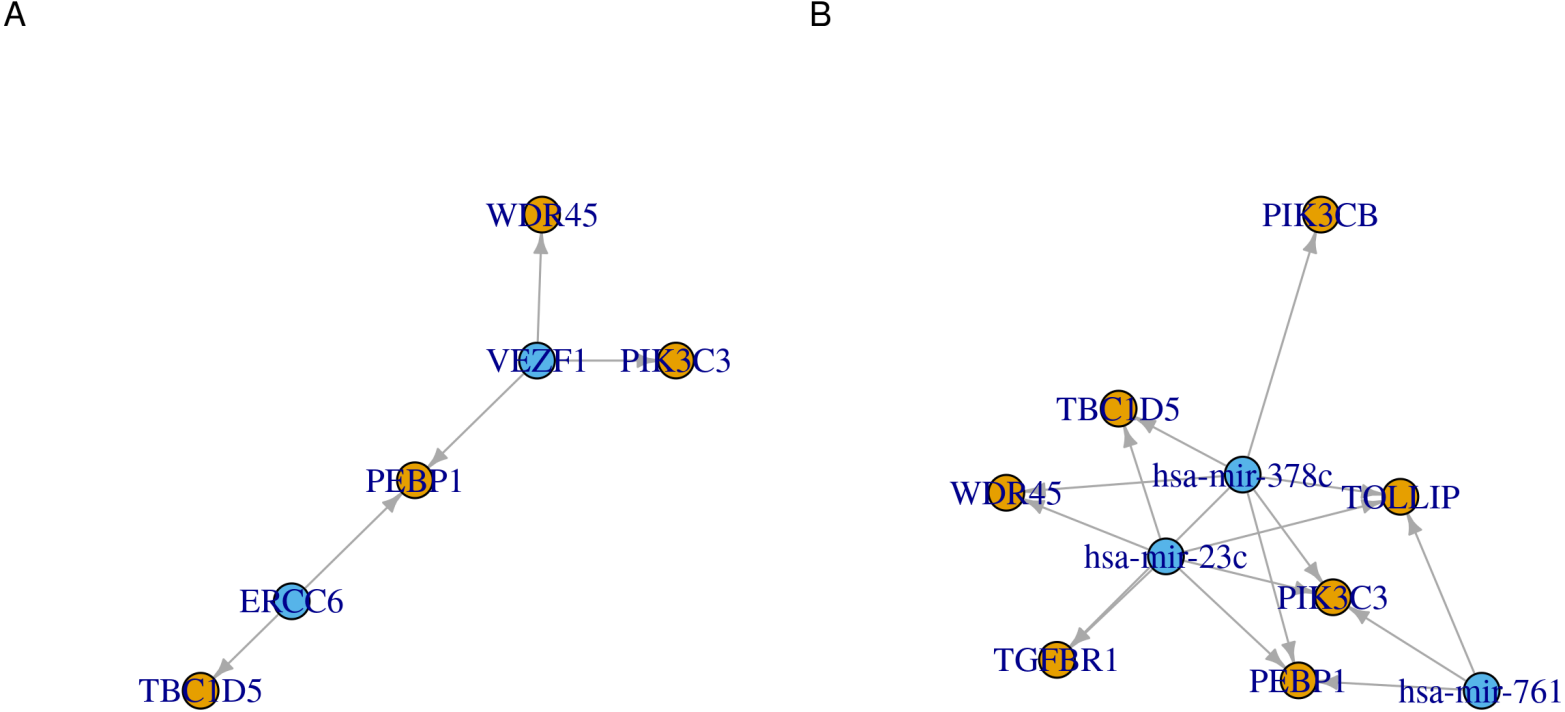
Network representation of transcription factor/microRNA targets. Directed graphs of (A) transcription factors or (B) microRNAs and their identified gene targets. Each of the transcription factors/microRNA is represented as a node. Nodes are connected by an edge when a gene is a target for the regulator. The direction of the edges is always from the regulators (transcription factor or microRNA, blue) to the targets (genes, orange).

Another way to visualize the identified regulations is to use network graphs. In Fig. 3, we used genes, transcription factors and microRNAs as graph nodes. The edges between the nodes were mapped to the co-expression correlations. In more denser graphs, clustering and node influence can be identified. In this case, hsa-mir-378c and hsa-mir-23c, having the highest number of edges, are expected to exert a higher influence on the network. This functionality can serve as both visualization method and an interface to other network analysis workflows.

**Figure 4:**
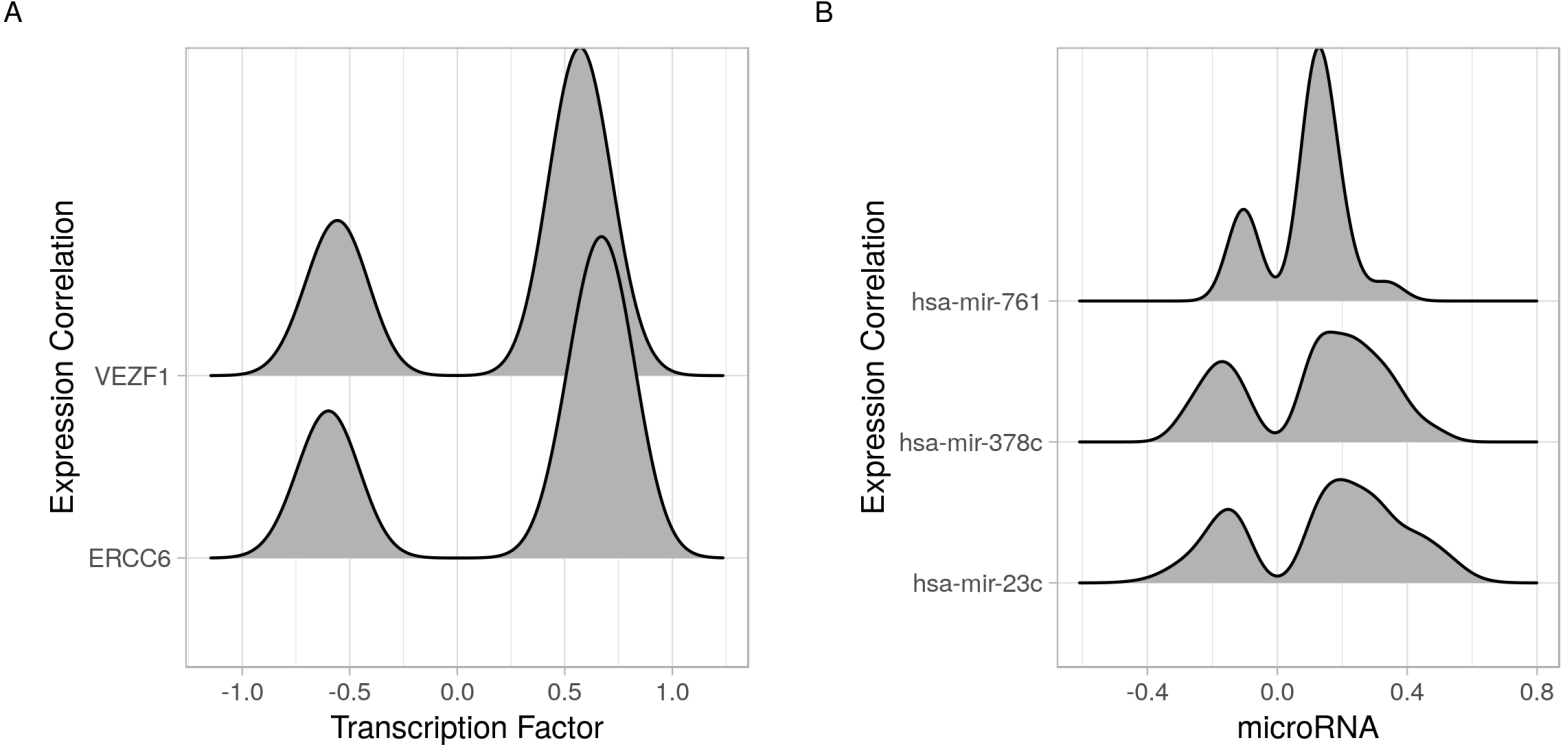
Joy plots of the density of transcription factor/microRNA expression correlations with all known genes. Density estimate for the expression correlations between target genes and (A) transcription factors (VEZF1 and ERCC6) and (B) microRNAs (hsa-mir-761, hsa-mir-378c, and hsa-mir-23c). The plot shows all possible expression correlation values (−1 to 0) on the *x*-axis and the probability of the realized value laying around it as the height, *y*-axis.

**Figure 5:**
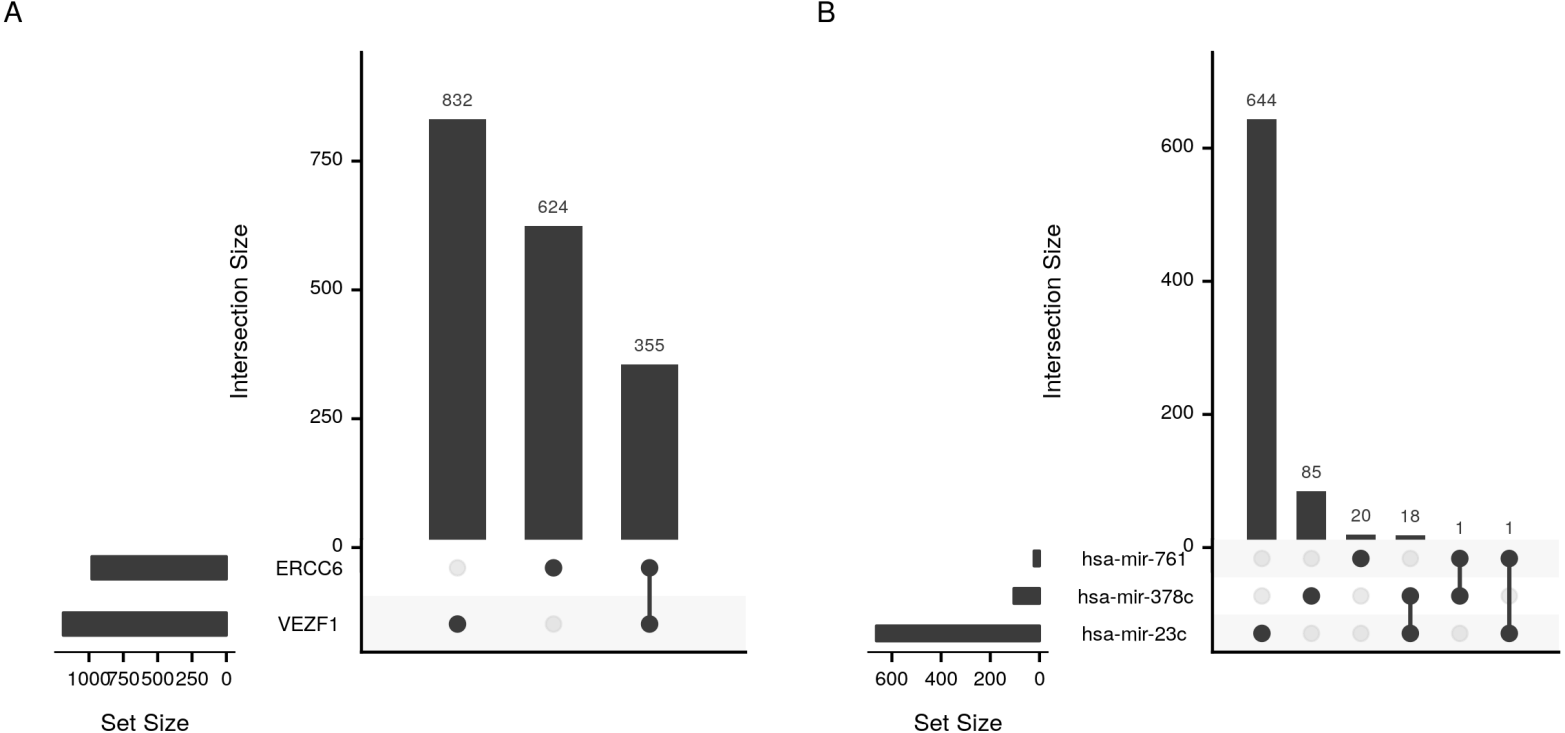
Upset plot of the intersection of transcription factor and microRNA target sets. Sizes and intersections of the sets of targets of (A) transcription factors (VEZF1 and ERCC6) and (B) microRNAs (hsa-mir-761, hsa-mir-378c, and hsa-mir-23c). The plot is divided into two parts; set size and intersection size. The size of each set of targets (horizontal) or intersect (vertical) is shown as bars. The intersect of two sets is indicated by a line connecting the two dots corresponding to the sets.

In addition to identifying regulatory relations of a set of genes of interest, cRegulome can be used to learn about the global role of certain regulators in different types of cancer. The two identified transcription factors, and to lesser extent the microRNAs, appeared to have both positive and negative regulatory roles in prostate cancer (Fig. 4). When this pattern was investigated in other types of cancer, both transcription factors tended towards the positive regulation (Fig. S1). In Fig. 5, we showed that while the two transcription factors share a significant number of target genes, the microRNAs do not. Although the shared number of targets between the two factors vary, it remains significant in several other cancer types.

### Code for reproducing the examples in the article

In this section, we discussed the steps of a typical cRegulome database query, data filtering and result visualization. The full code for reproducing this analysis was attached to this article (Additional File 1) and is available online at https://github.com/BCMSLab/cregart.

First, we start by installing the package, loading the required libraries and downloading the database file in an R session.

 
 
# Load  required  libraries 
library(RSQLite) 
library(cRegulome) 
library(ggplot2) 
 
# Installing  cRegulome 
install.packages(’cRegulome’)                       # stable version 
devtools::install_github(’ropensci/cRegulome’)   # development  version 
    


 
 
# Downlaod  database  file 
if(!file.exists(’cRegulome.db’)) { 
   get_db(test = FALSE, 
           destfile = ’./cRegulome.db’) 
} 


 
 
# Alternatively: the  database  file  can be  downloaded  using wget 
wget  https://s3-eu-west-1.amazonaws.com/pfigshare-u-files/9537385/ 
     cRegulome.db.gz 
gunzip  cRegulome.db.gz    

Second, we queried the database for the expression correlation of two transcription factors (ERCC6 VEZF1) and three microRNAs (hsa-mir-23c, hsa-mir-378c and hsa-mir-761). and limited the output to the nine genes of interest.

 
 
# Identify  target  genes 
targets  <- c(’PEBP1’, ’PIK3C3’, ’PIK3CB’, ’TBC1D25’, ’TBC1D5 
    ’, ’TOLLIP’, ’WDR45’, ’WIPI1’, ’TGFBR1’) 
# Query  mir  with  targets = False 
conn  <- dbConnect(SQLite(), ’./cRegulome.db’) 
 
mir  <- get_mir(conn, 
                  mir = c(’hsa-mir-23c’, ’hsa-mir-378c’, ’hsa- 
                      mir-761’), 
                  study = ’PRAD’, 
                  targets = targets) 
 
tf <- get_tf(conn, 
                tf = c(’ERCC6’, ’VEZF1’), 
                study = ’PRAD’, 
                targets_only = TRUE, 
                targets = targets) 
 
dbDisconnect(conn)    

The code to generate Figure 2:

 
 
ob <- cTF(tf) 
 
p1 <- cor_plot(ob) + 
   theme(legend.position = ’top’, 
          legend.direction = ’vertical’) + 
   scale_size_continuous(breaks = c(.5, .65)) + 
   labs(x = ’’ ’’, y = ’’ ’’) 
 
ob <- cmicroRNA(mir) 
 
p2 <- cor_plot(ob) + 
   theme(legend.position = ’top’, 
          legend.direction = ’vertical’) + 
   scale_size_continuous(breaks = c(.1, .3)) + 
   labs(x = ’’ ’’, y = ’’ ’’)    

The code to generate Figure 3:

 
 
ob <- cTF(tf) 
 
p1 <- cor_igraph(ob, directed = TRUE) 
 
ob <- cmicroRNA(mir) 
 
p2 <- cor_igraph(ob, directed = TRUE)    

Next, we constructed a query to find all targets of the five regulators in prostate cancer.

 
 
# Query  the  database 
conn  <- dbConnect(SQLite(), ’./cRegulome.db’) 
 
all_tf <- get_tf(conn, 
                     tf = c(’ERCC6’, ’VEZF1’), 
                     study = ’PRAD’, 
                     targets_only = TRUE) 
 
all_mir  <- get_mir(conn, 
                       mir = c(’hsa-mir-23c’, ’hsa-mir-378c’, ’ 
                           hsa-mir-761’), 
                       study = ’PRAD’, 
                       targets_only = TRUE) 
 
dbDisconnect(conn) 
    

The code to generate Figure 4:

 
 
ob <- cTF(alltf) 
 
p1 <- cor_joy(ob) + 
   labs(x = ’Transcription  factor’, 
         y = ’Expression  Correlation’) 
 
ob <- cmicroRNA(allmir) 
 
p2 <- cor_joy(ob)+ 
   labs(x = ’microRNA’, 
         y = ’Expression  Correlation’)    

The code to generate Figure 5:

 
 
ob <- cTF(alltf) 
 
cor_upset(ob) 
 
ob <- cmicroRNA(allmir) 
 
cor_upset(ob)    

## Discussion

cRegulome is an R package to access and visualize the expression correlation of gene regulatory elements (transcription factors and microRNAs) in cancer. The package provides a unified interface for two databases of integrative analyzes based on the TCGA data. In this article, we introduced the package functionality and a use-case from the published literature. Here, we further discuss using cRegulome for identifying common regulators of a gene set related to prostate cancer progression and refer to the limitations and the future development of the package.

In this article, we present a use case of cRegulome in which we investigated the common regulators of a group of co-expressed genes in prostate cancer. We found that two transcription factors (VEZF1 and ERCC6) and three microRNAs (hsa-mir-761, hsa-mir-378c, and hsa-mir-23c) simultaneously regulate PEBP1 and one or more of autophagy and EMT gene products. We previously found that this set of gene products were co-expressed and possibly functionally related during prostate cancer progression (Ahmed et al., 2018a). The common regulators may explain this correlation as genes that share regulators are expected to have similar expression patterns (Yu et al., 2003). Each regulation relationship occurred at least twice in other cancer types (Table S1). This might show how prevalent these regulators in cancer development. At the same time, it stresses the fact of regulation specificity to tissue and conditions (Sonawane et al., 2017; Chatterjee & Ahituv, 2017).

RTCGA, TCGAbiolinks and TCGAretriever R packages give several ways to access data from TCGA and other similar resources (Kosinski & Biecek, 2016; Colaprico et al., 2016; Fantini, 2016). caOmicsV can visualize multidimensional data from multiple cancer genomics resources (Zhang, 2018). Moreover, the datasets can be stratified by clinical (phenotype) data. mmultiOmicsViz can calculate and visualize correlations between two datasets that share genomic locations, but users has to provide the two datasets as input (Wang, 2018). cRegulome is concerned with accessing and visualizing integrative analyzes of gene regulation in cancer rather than the processed data on which it is based. In part, this reduces the time, and the effort needed to reproduce such analyzes.

Although we didn’t show in this article nor provided a mechanism in cRegulome for integrating transcription factor and microRNA data, it should be possible at least in principle simply by combining the output of two separate queries or their graphs. We used similar naming conventions for gene and study IDs which would make the combining of the two queries seamless. Users should consider the fact that the two types of data come from two different sources although the gene expression data is the same in both cases. Integrating transcription factors and microRNA target regulation can be useful as both regulators can be part of the same transcriptional complex (Martinez & Walhout, 2009). The direction of the correlation could add an extra layer of complexity as to determining whether the two factors agree or oppose regarding their regulatory potential of a certain gene.

Regulome elements that influence the gene expression are not only transcription factors and microRNA. Other elements include non-coding RNAs and DNA-binding proteins (Maston, Evans & Green, 2006). These elements as well as others modulate the behavior of cells in both physiological and pathological conditions (Chatterjee & Ahituv, 2017). Ideally, a comprehensive investigation of the gene regulation of a biological phenomena or a disease state should include all elements. The availability of data limits however what can be done at any certain study. For example, the TCGA project provides expression data for coding and non-coding gene expression as well as protein quantification that can be used in integrative analysis to that end. Projects like ENCODE and modENCODE provide a great resource for studying DNA binding elements although not focusing on cancer (The ENCODE Project Consortium, 2004; Roy et al., 2010). Going forward, other resources can be provided access to through cRegulome so that a more comprehensive study of gene regulation element can be done in an easily reproducible way.

## Conclusion

cRegulome provides programmatic access to the reglome (microRNAs and transcription factors) expression and their correlations with target genes in cancer. Here, we introduced an R package to obtain a local instance of Cistrome Cancer and miRCancerdb databases and provide objects and methods to interact with and visualize the correlation data.

##  Supplemental Information

10.7717/peerj.6509/supp-1 Figure S1 Expression correlation of common transcription factors with their target genes in all cancer studies

10.7717/peerj.6509/supp-2 Table S1 Repeated common regulatory interactions in other cancer studies

10.7717/peerj.6509/supp-3 Supplemental Information 1 Code

## Additional Information and Declarations

### Data Availability

The following information was supplied regarding data availability: GitHub: https://github.com/ropensci/cRegulome.
