# Supplementary material for: cRegulome: an R package for accessing microRNA and transcription factor-gene expression correlations in cancer
Source: PeerJ. 2019 Mar 8;7:e6509. doi: 10.7717/peerj.6509 (PMC6410695; doi:10.7717/peerj.6509)
Supplement: Figure S1 [file peerj-07-6509-s001.pdf]

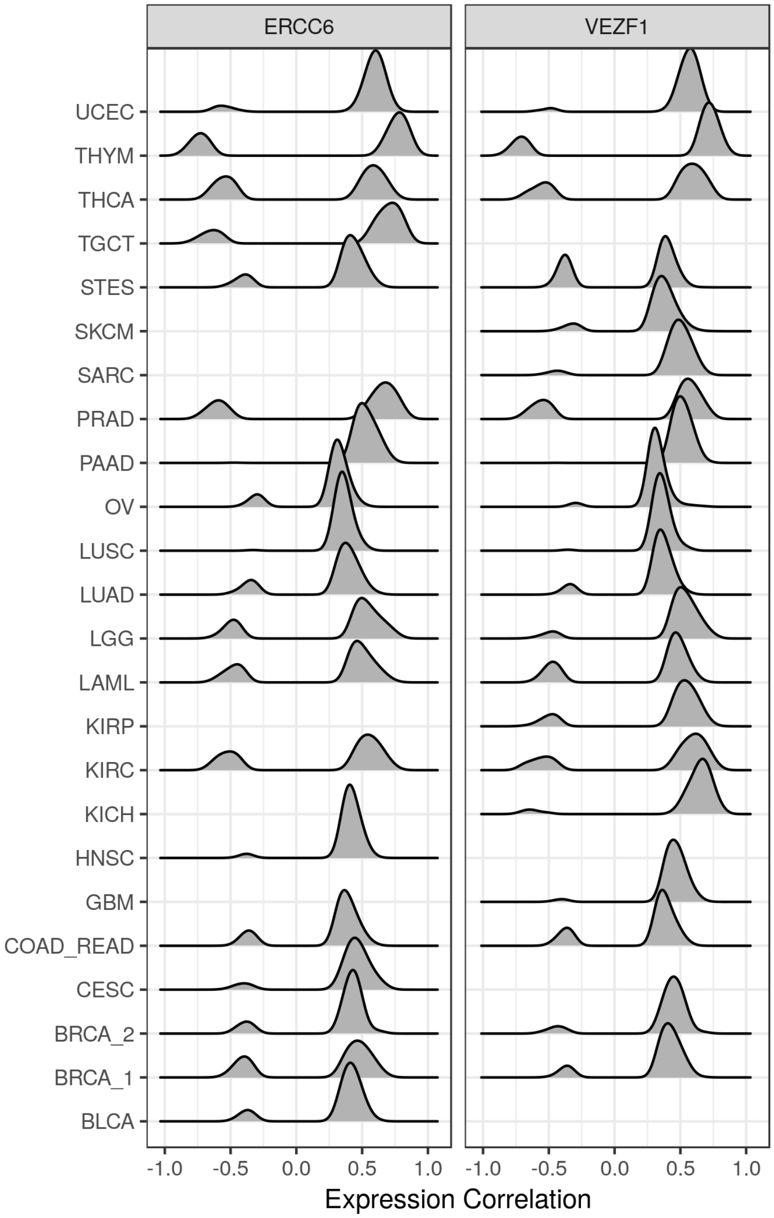

Figure S1: **Expression correlation of common transcription factors with their target genes in all cancer studies.** The plot shows all possible expression correlation values (-1 to 0) between the transcription factor and its targets in all cancer studies on the x-axis and the probability of the realized value laying around it as the height, y-axis.
