# Supplementary material for: cRegulome: an R package for accessing microRNA and transcription factor-gene expression correlations in cancer
Source: PeerJ. 2019 Mar 8;7:e6509. doi: 10.7717/peerj.6509 (PMC6410695; doi:10.7717/peerj.6509)
Supplement: Table S1 [file peerj-07-6509-s002.pdf]

Table S1: **Repeated common regulatory interactions in other cancer studies.**

TF	Gene	N	TCGA Studies
ERCC6	TBC1D5	15	BLCA, BRCA, CESC, COAD, KIRC, LAML, LGG, LUAD, OV, PAAD, PRAD, STES, TGCT, THCA, THYM
	PEBP1	2	PRAD, THCA
VEZF1	TBC1D5	9	BRCA, COAD, KIRC, KIRP, LAML, STES, THCA, THYM, UCEC
	PIK3C3	8	BRCA, GBM, KIRC, LGG, PAAD, PRAD, SARC, THCA
	WDR45	3	KIRC, PRAD, THYM
	PEBP1	2	LGG, PRAD
